# LINKS BETWEEN PARTNER INTERACTIONS, EMPATHY, AND EVERYDAY PHYSIOLOGICAL SYNCHRONY IN OLDER COUPLES

**DOI:** 10.1093/geroni/igz038.1629

**Published:** 2019-11-08

**Authors:** Theresa Pauly, Victoria I Michalowski, Johanna Drewelies, Denis Gerstorf, Maureen C Ashe, Kenneth M Madden, Christiane A Hoppmann

**Affiliations:** 1 University of British Columbia, Vancouver, Canada; 2 University of British Columbia, Vancouver, British Columbia, Canada; 3 Humboldt-Universität Zu Berlin, Berlin, Berlin, Germany

## Abstract

Romantic partners exhibit dyadic covariation (synchrony) in physiological parameters. This study aims to link everyday cortisol synchrony to daily partner interactions and empathy. We conducted coordinated multilevel analysis using data from two independently collected samples of older couples (Study 1: N = 85 couples, aged 60-87 years; Study 2: N = 77 couples, aged 66-85 years) who completed questionnaires and provided salivary cortisol samples 5 to 7 times daily for 7 days. Cortisol levels were significantly correlated among partners in both studies. Cortisol synchrony was higher when partners were present (Study 1), and when partner interactions involved feeling understood and valued (Study 1) and seeking help or closeness (Study 2). Higher cortisol synchrony was further related to greater empathic accuracy (Study 1) and greater empathy (Study 2). Thus, social bonding processes and the ability to consider other’s thoughts and feelings may be intertwined with physiological synchrony in everyday life.

